# The genetic structure of populations of *Isthmiophora melis* (Schrank, 1788) (Digenea: Echinostomatidae). Does the host’s diet matter?

**DOI:** 10.1186/s13071-023-05811-3

**Published:** 2023-06-07

**Authors:** Grzegorz Zaleśny, Gerard Kanarek, Ewa Pyrka, Marta Kołodziej-Sobocińska, Andrzej Zalewski, Joanna Hildebrand

**Affiliations:** 1 Department of Invertebrate Systematics and Ecology, Institute of Environmental Biology, Wrocław University of Environmental and Life Sciences, Kożuchowska 5B, 51-631 Wrocław, Poland; 2 Ornithological Station, Museum and Institute of Zoology, Polish Academy of Sciences, Nadwiślańska 108, 80-680 Gdańsk, Poland; 3 Department of Parasitology, Faculty of Biological Sciences, Wrocław University, Przybyszewskiego 63, 51-148 Wrocław, Poland; 4 Mammal Research Institute, Polish Academy of Sciences, Stoczek 1, Białowieża, Poland

**Keywords:** *Isthmiophora melis*, Digenea, Host–parasite relationship, Genetic population structure, Rodents, American mink

## Abstract

**Background:**

Here we provide a comparative analysis of the genetic structure of populations (based on *nad1* mtDNA) of *Isthmiophora melis* isolated from the American mink (*Neogale vison*), an introduced invasive species, commonly occurring in the territory of Poland, and from the striped field mouse (*Apodemus agrarius*).

**Methods:**

A total of 133 specimens of *I. melis* were obtained from naturally infected *N. vison* collected from six localities in Poland (108 samples) and 25 individuals of *I. melis* from *A. agrarius*. All sequences of the *nad1* gene obtained during the present study were assembled and aligned. The standard statistics for haplotype composition, i.e., the number of haplotypes, haplotype diversity, nucleotide diversity, and average number of nucleotide differences, were calculated. Haplotype analysis and visualization of haplotype frequency among populations were performed using a median-joining network.

**Results:**

Based on the samples collected from different localities in Poland, our study revealed that the overall genetic diversity of *I. melis* isolated from the American mink and of the striped field mouse do not differ significantly. The median-joining network showed that the three main haplotypes are in the centre of a star-like structure, with the remaining haplotypes as the satellites, reflecting the recent expansion of the populations.

**Conclusions:**

The overall genetic diversity of *I. melis* isolated from the American mink and striped field mouse reveals a high level of homogeneity. Moreover, regional differences in the food composition of the definitive hosts play an important role in shaping the genetic structure of the trematode populations.

**Graphical Abstract:**

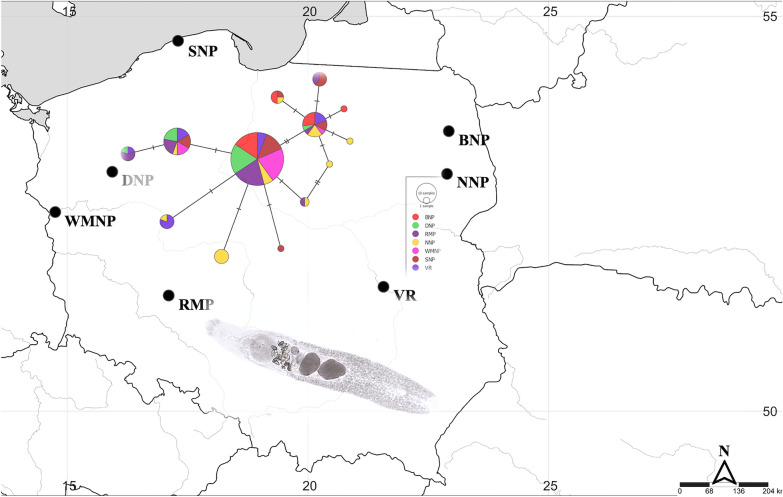

## Background

The genus *Isthmiophora* Lühe, 1909 constitutes a group of 27- and 29-collar-spined echinostomes [1]. The members of the genus are common parasites of many mammal species, mainly carnivores and rarely rodents or humans, serving as definitive hosts [2]. The most common species, and at the same time a type species of this genus, is *Isthmiophora melis* (Schrank, 1909), which occurs in Europe, Asia, and North America (e.g., [3–6]). According to Radev et al. [2], at least 30 species serve as a definitive host for this parasite, including badger *Meles meles* L., 1758, being a type host, American mink *Neogale vison* (Schreber 1777), otter *Lutra lutra* (L., 1758), and other carnivores. In 1988, Esch et al. [7] introduced the allogenic/autogenic species concept to correlate helminth colonization processes and the structure of parasite communities among freshwater fishes. According to that concept, the life cycle of *I. melis* is allogenic (i.e., completed in semi-aquatic conditions, contrary to the autogenic one which takes place in an aquatic environment) and includes two intermediate hosts, the pulmonate freshwater snail *Lymnaea stagnalis* (L., 1758) and a freshwater vertebrate (amphibian or fish). Later, in 2013, Blasco-Costa and Poulin [8] performed a meta-analysis proving that the life cycle type (allogenic vs autogenic) accurately predicts the population genetic structure in helminth communities.

In our previous work [4] we showed that the morphology of *I. melis* is strongly affected by host species and that this parasite reveals a high level of host-induced morphological variability. According to Kanev et al. [9], adult echinostomes can survive in the definitive host from 3 weeks up to more than 1 year, depending on the parasite species. The previous observation of the morphology of specimens that infect long-lived carnivores (with a life expectancy accounting for several years) compared to short-lived (up to a few months) rodents allows us to put forward the hypothesis that phenotypic variation may correlate with the lifespan of the host species or crowding effect resulting from the parasite density [4]. It is likely that some aspects of host biology, i.e., physiology or biochemical processes, also affect the morphological variability of digeneans; however, they are underestimated and should be carefully examined. According to Radev et al. [2], amphibians and fish serve as second intermediate hosts, which indicates that the rodent infected with *I. melis* must have eaten either the amphibian or the fish. Contrary to the data on *Isthmiophora hortensis* (Asada, 1926), there is no strong evidence in the literature that *I. melis* exploits fish as a second intermediate host. Nevertheless, such a scenario does not seem unrealistic under certain conditions at periodically drained fish ponds, which are present, for instance, in the Milicz Ponds Nature Reserve (RMP), when the significant drop in the water level may increase the availability of an atypical food source for rodents, such as tadpoles or small fish. On the other hand, quite a different scenario, in which the rodent feeds on infected snails, thus being in fact the second intermediate host within which the parasite completes its life cycle, cannot be ruled out. This hypothesis assumes that the neotenic forms of *I. melis* can occur in rodents [4]. In fact, truncation of the life cycle enabling the development of progenetic metacercariae which exploit the second intermediate host as a definitive host has been reported in a wide range of digenean taxa, but never in Echinostomatidae [10]. However, assuming the latter scenario, it would be expected that the *I. melis* population in rodents is genetically less diverse than the populations from the typical definitive hosts. Considering these facts, the proper selection of molecular markers should allow the depiction of these differences. Molecular identification of *I. melis* based on the small subunit (SSU) and large subunit (LSU) of ribosomal DNA (rDNA) would make it possible to assign specimens to genus level only, but using less conservative markers, such as the internal transcribed spacer 1 (ITS1)/ITS2 of rDNA and *cox1* of mitochondrial DNA (mtDNA), would provide a correct match to the species [4]. However, a few works have investigated the usefulness of ITS sequences versus mitochondrial markers in the taxonomy of Echinostomatidae [11, 12]. The discrepancy between sequences varied from 2.2% for ITS rDNA and 8% for *cox1* to 14% for *nad1* mtDNA. The authors concluded that the mtDNA *nad1* marker was the best for *Echinostoma* species due to its greater pairwise divergence relative to other markers, and considered it the most informative marker in studies of relationships within Echinostomatidae [11, 13].

Taking into account a relatively low scale of intraspecific genetic variation of some representatives of Echinostomatidae, in this work we analyse the genetic structure of the *I. melis* population based on samples collected in different regions of Poland. Our studies are based on specimens of *I. melis* isolated from the American mink (*N. vison*), an invasive species, and from the striped field mouse (*A. agrarius*), both commonly occurring in Poland. Moreover, we test the assumption on the correlation between the host’s diet and the genetic structure of the parasite population. It is likely that the genetic structure of the *I. melis* population is affected by the host diet, i.e., the greater share of amphibians and fish in the diet will promote an increase in the genetic diversity of the parasite population.

## Methods

### Parasite sampling

**Fig. 1:**
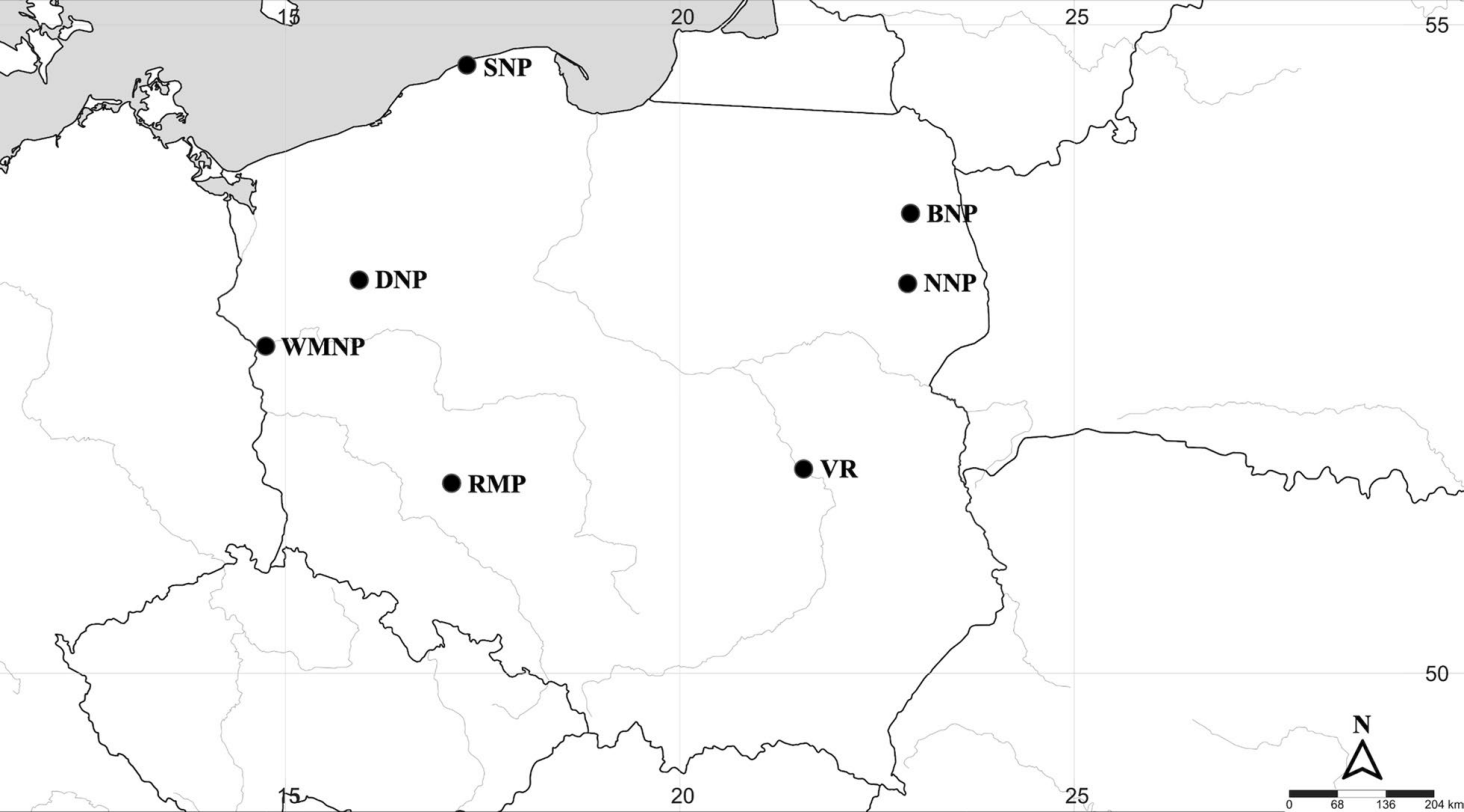
Sampling sites of individuals of *Isthmiophora melis* in Poland. Study sites are marked with dots: WMNP—Warta Mouth National Park; DNP—Drawa National Park; SNP—Słowiński National Park; BNP—Biebrza National Park; NNP—Narew National Park; VR—Vistula River; RMP—Milicz Ponds Nature Reserve (the map was created using SimpleMappr—https://www.simplemappr.net)

A total of 108 specimens of *I. melis* (each isolate came from a different host individual) were obtained from naturally infected hosts of the American mink from six localities in Poland: Biebrza National Park (BNP), Warta Mouth National Park (WMNP), Narew National Park (NNP), Drawa National Park (DNP), Słowiński National Park (SNP), and Vistula River (VR, Fig. 1). The material was collected during American mink eradication carried out by the staff of national parks or bird conservation organizations as part of various bird conservation projects (e.g. [14]. Thus, based on Resolution No. 22/2006 of the National Ethics Committee for Animal Experiments, no separate approval from the local ethics committee for animal experimentation was needed to obtain parasites from carcasses and to carry out the study [15]. Additionally, 25 individuals of *I. melis* (derived from 10 host individuals) were obtained from the striped field mouse in the RMP (Fig. 1), one of the largest pond complexes in Europe (the reserve was established on an area of over 6000 hectares due to its importance as a habitat and breeding ground for water birds). The material was collected based on permission 46/2008 issued by the Second Local Commission for Animal Experiments.

### DNA extraction, amplification, and sequencing

Specimens were stored in 70% ethanol (at −20 °C) prior to further processing. Before DNA isolation, specimens were washed twice in TE buffer in order to remove ethanol. A single specimen of *I. melis* was used for each DNA extraction. DNeasy Blood & Tissue Kits (Qiagen) were used to isolate DNA, following the manufacturer’s protocol. A partial fragment of the mitochondrial gene coding the nicotinamide adenine dinucleotide dehydrogenase subunit 1 (*nad1*) was amplified during the polymerase chain reaction (PCR). PCR was performed in a total volume of 15 µl (4 µl H_2_O, 7.5 µl KAPA2G Robust PCR Kit, 0.75 µl of each primer, 2 µl of template DNA isolate). The amplification program following PCR comprised initial denaturation at 95 °C for 5 min, followed by 35 cycles with 30 s denaturation at 94 °C, 20 s for primer annealing at 48 °C, and 45 s for primer extension at 72 °C, with a final extension step at 72 °C for 8 min. PCR was performed using the following primer pairs: forward NDJ11 (5′-AGA TTC GTA AGG GGC CTA ATA-3′) and reverse NDJ2a (5′-CTT CAG CCT CAG CAT AAT-3′) [16]. PCR products were purified using the Exo-BAP Kit (EURx).

### Analysis of genetic population structure of *I. melis*

All sequences (*n* = 133) of the *nad1* gene obtained during the present study were assembled and aligned in Geneious v9. All standard statistics for haplotype composition, i.e., the number of haplotypes (H), haplotype diversity (Hd), nucleotide diversity (Pi), and the average number of nucleotide differences (K), were calculated using DnaSP v5 [17]. Haplotype analysis and visualization of haplotype frequency among populations were performed in PopArt v1.7 [18] using a median-joining network. Analysis of molecular variance (AMOVA), along with the estimation of genetic distances (*F*_ST_), was carried out in Arlequin version 3.5 [19].

### The relationship between *N. vison* diet and genetic structure of *I. melis* populations

In 2019, Chibowski et al. [20] analysed the diet of *N. vison* from four localities, i.e., DNP, NNP, BNP, and WMNP. Their research was based on the same material (i.e., American mink) that was used for this study. Thus, it was possible to correlate the information on diet and the genetic structure of *I. melis*. The diet of *N. vision* is varied and includes representatives of many different groups of vertebrates (including rodents, birds, amphibians, and fish), but only amphibians and fish play a role in the transmission of *I. melis*. In the case of *A. agrarius*, we assumed that the infection with *I. melis* was a result of consuming either fish or amphibian specimens. Therefore, we treated the population of *I. melis* from the RMP as a reference for the mink population.

## Results

**Fig. 2:**
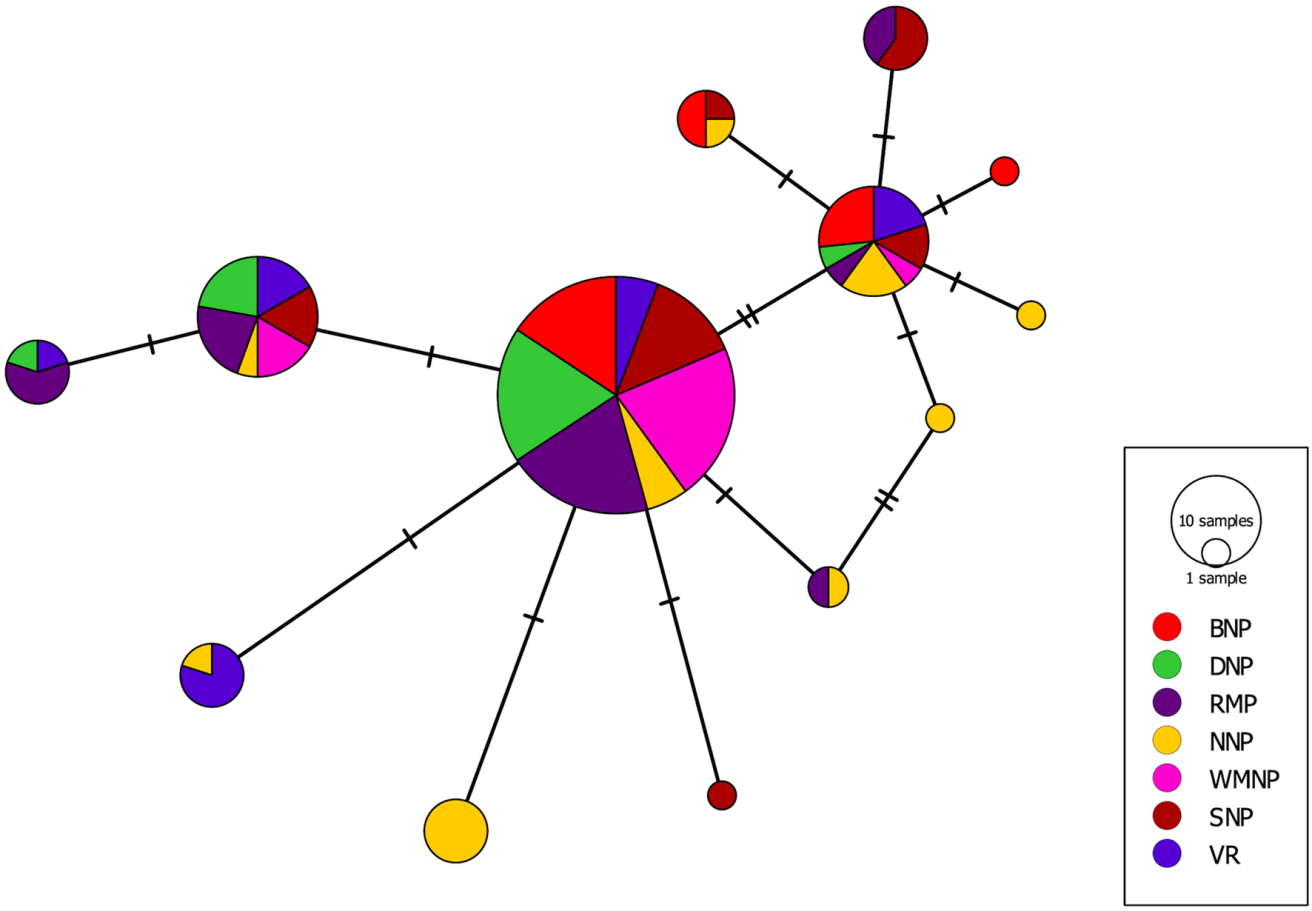
Median-joining network of samples of *Isthmiophora melis* built with the (*nad1*) gene

In total, 133 sequences (trimmed to 402 base pairs) of *I. melis* with 13 haplotypes were obtained from the analysed populations. The mean Hd was relatively high (0.690), with Pi attaining a very low level (0.003). Discrepancies were observed in the Hd values between populations; the lowest Hd was in WMNP (0.368), while the highest (0.876) was in NNP (Table 1). The main haplotypes (H1 and H2) were found in all populations, while H6 was present in six populations, being absent in NNP. Haplotypes H4, H11, H12, and H13 were reported only in BNP, NNP, NNP, and SNP, respectively. The median-joining network showed that the three main haplotypes are in the centre of a star-like structure, with the remaining haplotypes as the satellites (Fig. 2). Such shape reflects the recent expansion of the populations, which was also confirmed by the negative results of Tajima’s *D* test for all populations except BNP and VR (Table 1). The AMOVA (Table 2) showed that the differences between populations were much lower (6.85% of the total variation) than the observed differences of intra-population origin (93.15% of the total variation). The genetic distance between populations was also assessed using the *F*_ST_ values (Table 3), with results similar to the previously described parameters. The highest *F*_ST_ values were observed between DNP and NNP and between NNP and WMNP; in both cases the differences were statistically significant.

**Table 1:** Molecular characteristics of *nad1* haplotypes obtained from seven populations of *Isthmiophora melis* in Poland

Population	No.	S	H	Hd ± SD	Pi	K	Tajima’s *D*	Fu’s *F*_S_
BNP	18	4	4	0.595 ± 0.109	0.003	1.327	0.4227	0.536
DNP	19	4	4	0.509 ± 0.117	0.002	0.725	−1.0788	−0.787
NNP	18	8	9	0.876 ± 0.051	0.005	2.020	−0.4577	−3.665
WMNP	19	3	3	0.368 ± 0.125	0.001	0.491	−1.1630	−0.283
SNP	19	6	6	0.749 ± 0.086	0.004	1.684	−0.0612	−0.837
VR	15	5	5	0.829 ± 0.049	0.004	1.657	0.2619	−0.270
RMP	25	6	6	0.663 ± 0.092	0.003	1.313	−0.5189	−1.101
Overall	133	12	13	0.690 ± 0.039	0.003	1.380	−0.9497	−4.760

*BNP* Biebrza National Park, *DNP* Drawa National Park, *NNP* Narew National Park, *WMNP* Warta Mouth National Park, *SNP* Słowiński National Park, *VR* Vistula River, *RMP* Milicz Ponds Nature Reserve, *n* number of sequences, *S* segregating sites, *H* number of haplotypes, *Hd* haplotype diversity, *Pi* nucleotide diversity, *K* average number of nucleotide differences

**Table 2:** The results of AMOVA of *nad1* sequences of *Isthmiophora melis* derived from seven populations in Poland

Source of variation	*df*	Sum of squares	Variance components	Percentage variation
Among populations	6	4.657	0.0238 Va	6.85
Within populations	126	40.892	0.3245 Vb	93.15
Overall	132	45.549	0.3484	
Fixation index *F*_ST_	0.068

**Table 3:** *F*_ST_ pairwise comparison (with *F*_ST_ *P*-values) of *nad1* sequences of *Isthmiophora melis* derived from seven populations in Poland

	BNP	DNP	NNP	WMNP	SNP	VR	RMP
BNP	–	0.1351 ± 0.0339	< 0.02	0.0811 ± 0.0163	0.2252 ± 0.0311	0.1441 ± 0.0388	0.1441 ± 0.0388
DNP	0.0326	–	< 0.0001	0.8468 ± 0.0365	0.3153 ± 0.0434	0.6577 ± 0.0430	0.6577 ± 0.0430
NNP	0.1044	0.1649	–	< 0.0001	0.0270 ± 0.0139	< 0.02	< 0.02
WMNP	0.0489	−0.0349	0.2319	–	0.1261 ± 0.0242	0.1802 ± 0.0271	0.1802 ± 0.0271
SNP	0.0138	0.0133	0.0620	0.0623	–	0.0991 ± 0.0417	
VR	0.1051	0.1278	0.0328	0.0753	0.0405	–	0.0450 ± 0.0152
RMP	0.0295	−0.0211	0.1066	0.0232	−0.0186	0.0753	–

BNP—Biebrza National Park, DNP—Drawa National Park, NNP—Narew National Park, WMNP—Warta Mouth National Park, RMP—Milicz Ponds Nature Reserve. Above diagonal—*F*_ST_ *P*-values; below diagonal—*F*_ST_ values

**Fig. 3:**
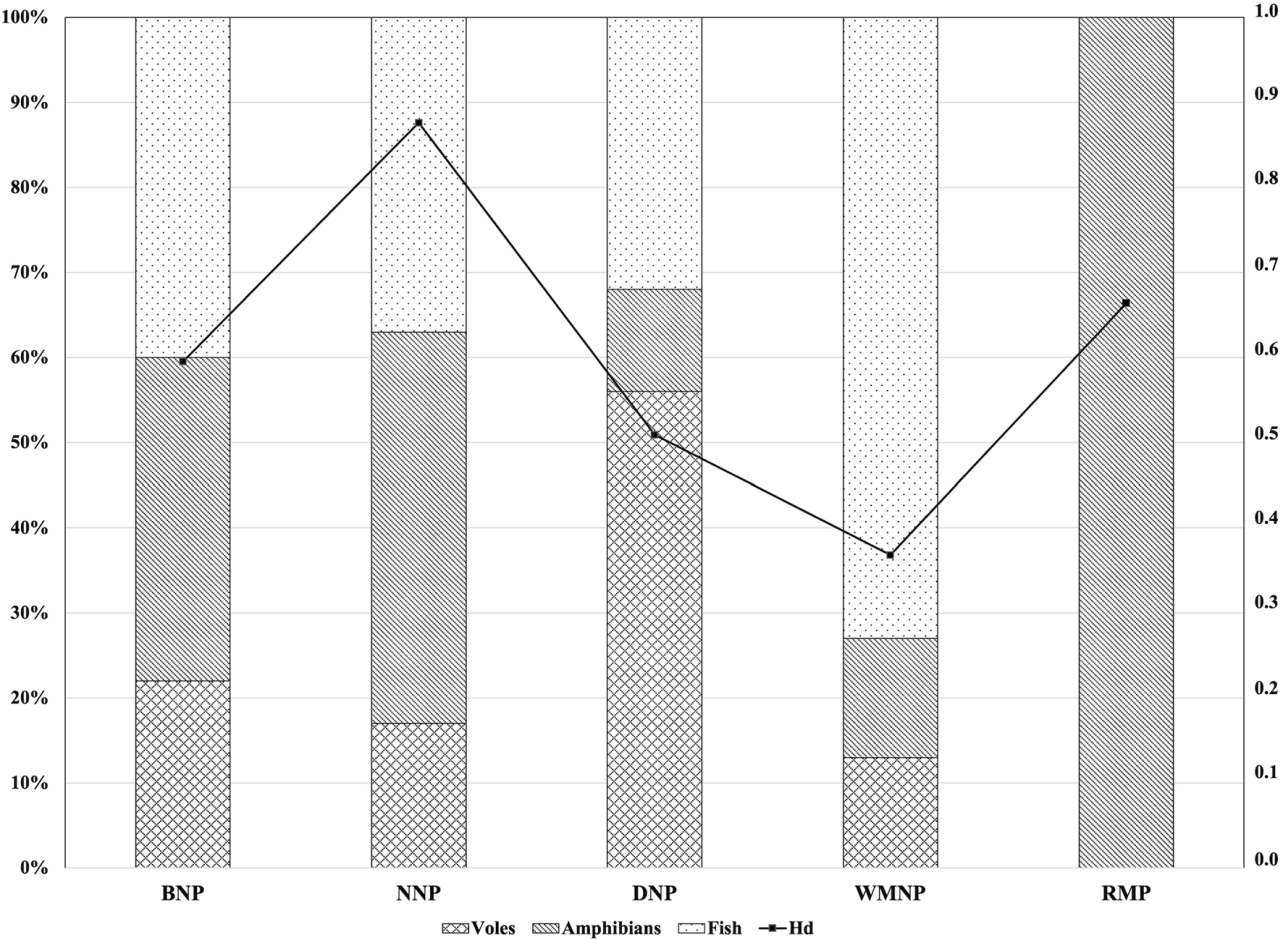
The relationship between host diet and haplotype diversity (Hd - the values provided on right y-axis) of *Isthmiophora melis*

In the next step, we check whether there is a relationship between the host's diet and the genetic diversity of the *I. melis* population. For this purpose, data from the study by Chibowski et al. [20] were used. The authors analysed the diet of *N. vison* from four localities, i.e., DNP, NNP, BNP, and WMNP. Consequently, this part of the results will be based on the material originating from the above-mentioned localities only, with reference to RMP. In the case of RMP, it was assumed that the only source of infection may be amphibians, which become an easily available source of protein for rodents at the time of periodic draining of the fish ponds. Therefore, it was assumed that the reference value for RMP is 100% (the only source of infection) and the Hd indices for the remaining populations were compared to the RMP. The Hd values were higher in the case of populations for which the amphibians constituted the main food source (NNP and BNP, 46% and 38%, respectively), and much lower in populations where rodents and fish predominated as food (Fig. 3).

## Discussion

Digeneans are one of the most unique and the most numerous groups of parasitic Platyhelminthes. Their life cycle is complex, with few parthenogenetic and a single hermaphroditic generation [21]. Due to the complexity of the life cycle, these parasites are frequently subject to various modifications to their life strategies. There are several ways that the life cycle can be modified [10]; however, modification usually leads to the narrowing of the host spectrum. We can come across the results of numerous studies in the literature on life cycle modifications in digeneans (e.g., [22–24]). In addition to these interesting alterations, it is also worth noting that the process of reproduction takes place both sexually (in the definitive host) and asexually (at the level of intermediate hosts) in these parasites. Therefore, if we associate these data with the previously mentioned features of digenean life strategies, we obtain the set of main factors that could affect the genetic structure of the given population. Moreover, the genetic structure of trematode populations also depends on the type of life cycle, i.e., allogenic (semi-aquatic) or autogenic (in the aquatic environment). According to Blasco-Costa and Poulin [8], trematodes with an autogenic life cycle express significantly more pronounced genetic structure than those leaving the aquatic environment in mammalian or bird hosts. *Isthmiophora melis* is among the trematodes with an allogenic life cycle, i.e., having the pulmonate freshwater snail *L. stagnalis* as the first intermediate host*,* and freshwater vertebrates (amphibians and fish), as the second intermediate host, while the sexual reproduction occurs in carnivorous mammals. Our results showed that the genetic structure of *I. melis* reflects the pattern typical for such organisms, i.e., reaching the high values of Hd, no statistically significant variation between populations, the negative results of Tajima’s *D* test, and star-like shape of haplotype network [25]. Therefore, it seems that the major factor affecting the genetic structure of *I. melis* is the biology of the host.

The striped field mouse is a non-synanthropic rodent species, feeding predominantly on plant food. However, some studies have shown that animal-derived components are also found in its diet (e.g., [26–28]). Studies carried out in urbanized areas of Warsaw and its surroundings reported a dominant share of representatives of invertebrates in the food of *A. agrarius*, and the presence of vertebrate remains was found in almost 5% of the rodents examined [27]. In the RMP, the water in the ponds is drained periodically, which is related to the carp production cycle, making tadpoles an easily available source of food for rodents (personal observation). The other analysed host, the American mink, is a species of mustelid which is native to North America and introduced to Europe. The diet of this species has been analysed in various studies using different methods, and its composition is likely to be strongly affected by extrinsic factors such as habitat, weather conditions, seasonality, or the abundance of potential prey (e.g., [20, 29–32]); nevertheless, the core components are rodents, fish and amphibians. Thus, this species falls among the most common definitive host of *I. melis*.

As we reported previously, the wide host range of *I. melis*, combined with the various aspects concerning body size or physiological conditions of its hosts (e.g., badger, mink vs striped field mouse), may cause the parasites to display a clearly visible phenotypic plasticity [4], or alternatively, the morphological discrepancies not being due to phenotypic plasticity, may result from life cycle modifications. In this study, we have revealed that molecular diversity at the intra-community level of rodent-derived *I. melis* can be even higher than in a typical host such as American mink (i.e., WMNP vs RMP) or can attain a similar value (i.e., BNP, DNP). Of course, these studies also could have been performed with the application of other markers, such as single-nucleotide polymorphism (SNP) or microsatellites. However, since the use of the *nad1* mtDNA marker showed intra- and inter-population differences, it can be concluded that the chosen methodology was sufficient. The material used in our study was also used in the research conducted by Chibowski et al. [20], where the authors performed an isotopic analysis of the diet of American mink obtained from four locations (BNP, DNP, WMNP, and NNP). This allowed us to compare the composition of the diet of the host and the genetic variability of *I. melis*. The results support the hypothesis that in trematodes with an allogenic life cycle, the biology of the host shapes the genetic structure of the parasites. When voles—i.e., not a potential source of *I. melis* infection—dominated the diet of American mink, the genetic diversity of the parasite was at a low level. In contrast, when the diet was dominated by amphibians and, to a lesser extent, fish, the intra-population genetic diversity of *I. melis* increased. Thus, regional differences in the host diet seem to affect the genetic structure of the parasite population. Similar results were observed in studies by Sharrard-Smith et al. [33], who evaluated the molecular phylogeny and distribution of the representatives of Opisthorchiidae in otters (*Lutra lutra*) and mink. The authors showed that differences in the phylogenies between the two species *Metorchis bilis* and *Pseudamphistomum truncatum* may suggest divergent demographic histories, possibly reflecting contrasting host diets. It is also worth emphasizing that the correlation between the host's diet and the presence of parasites is an important element necessary to understand the functioning of the food web in the ecosystem.

## Conclusions

Our study showed that the overall genetic diversity of *I. melis* isolated from the American mink and striped field mouse do not differ. The haplotype distribution analysis showed recent expansion of the populations, where the biology of the definitive host plays an important role in the genetic structure of the parasite. Finally, the results confirm that the genetic structure of the *I. melis* population is related to the diet composition of the definitive host.

## Data Availability

All data, including nucleotide sequences, are available upon request.
